# Celebrating five years of “Hematology without Borders” by the International Academy for Clinical Hematology (IACH)

**DOI:** 10.46989/001c.91538

**Published:** 2024-01-05

**Authors:** Mohamad Mohty, Arnon Nagler, Bipin Savani

**Affiliations:** 1 Service d’Hématologie Clinique et Thérapie Cellulaire Sorbonne Université, Saint Antoine Hospital and INSERM UMRs 938, Paris, France; 2 Chaim Sheba Medical Center, Tel Aviv University, Tel-Hashomer, Israel; 3 Division of Hematology and Oncology Vanderbilt University Medical Center, Nashville, Tennessee, USA

**Keywords:** IACH

The International Academy for Clinical Hematology (IACH) was conceived by an international group of physicians whose focus is to promote good clinical practice in the field of hematology. The IACH was launched in 2018 but, at that time, many colleagues were skeptical about the concept of using digital tools to spread and disseminate knowledge and education. The COVID-19 pandemic drove the whole planet into the “zoom era”, and immediately the IACH proved to be a visionary and a rapidly growing project. In addition to a couple of classical annual meetings, the current IACH portfolio includes a variety of educational “virtual” activities, allowing for an impressive reach to hematologists and other allied health care professionals in more than 130 different countries across the globe. Several thousands of attendees have followed and taken advantage of an average of 2 activities per week in 2023. The IACH managed to fulfill the unmet needs not covered by other sister organizations, and attracted a broader audience for the benefit of hematology everywhere. Such expansion and massive reach will continue to grow in the near future, given the pathophysiological knowledge and therapeutic revolution we are witnessing in hematology. There is, indeed, a growing and urgent global demand for continuing education of hematologists, comprising clinical skills for patient management, optimization of real-life treatment procedures, and in the performance of routine and comprehensive state of the art care in the various fields of hematology.

Currently, and based on the number of drugs being approved every year by regulatory agencies worldwide, hematology is likely among the most dynamic medical specialties. The development of new agents (small molecules, targeted therapies, chemo-free regimens, immunotherapy, etc.) is accompanied by a crucial need for sharing experiences to improve the quality of patient treatment and care through the development of professional competencies and excellence. In order to attain these objectives, cooperation is needed between health professionals worldwide under a common umbrella to promote and share health care capacities.

Within this background, and since its inception, the IACH aimed to create a comprehensive intellectual, operational, and agile environment for acquisition of knowledge, skills, clinical judgement, and attitudes essential for the best practice of clinical hematology. To achieve its goals, the IACH adopted the highest standards of quality supported by surveys of its members and friends, and a continuous close contact with colleagues who have their “boots on the ground” to sense the educational needs of the community. The high level of the IACH educational activities allowed it to proudly become an official provider of Continuing Medical Education-Continuing Professional Development (CME-CPD) of the European Board for Accreditation in Hematology (EBAH). EBAH awards accreditation of highly reputable scientific and medical associations and societies as education providers, after strict reviewing and monitoring, if they consistently show that they follow the standards and meet the requirements for delivering independent CME-CPD that accelerates learning, change, and improvement in healthcare.

The growth of the academy will not stop at its 5^th^ anniversary, and the future looks bright, because the IACH nurtures the ‘maverick thinking’. Thus, in 2024 and beyond, we will continue to positively surprise the clinical hematology community, as we would like to stay ahead of the game and never miss a step. New educational initiatives for pharmacists and patients are in the pipeline. Further, audacity and risk taking are part of the IACH DNA, because they are mandatory attributes to staying at the top. We will continue to avoid risk aversion, encourage unregimented thinking, and push for adaptability and agility. The IACH is your open access hub for education in clinical hematology, and all IACH members, friends and supporters have the opportunity to indicate their interest to join and contribute to the different scientific activities. We need all energies to accomplish our vision, and to pioneer these transformative and impactful groundbreaking changes.

In this new year of Clinical Hematology International, the official journal of the IACH, you will read some high-level clinical reviews and original reports from different parts of the globe. Promoting the concept of “Hematology without Borders”^©^, the IACH is about diversity, equity, inclusion, innovation, inspiration, open and collaborative mindset and, most importantly, improving patients’ outcome, because patients matter to us wherever they live. Providing high-quality education and dissemination of knowledge is the foundation of high-level clinical care, and good care is a pre-requisite for curing more and more patients. Cure is the goal. We hope you will continue to enjoy the IACH activities.

